# Angiotensin II type 1 receptor expression in ovarian cancer and its correlation with tumour angiogenesis and patient survival

**DOI:** 10.1038/sj.bjc.6602961

**Published:** 2006-01-24

**Authors:** K Ino, K Shibata, H Kajiyama, E Yamamoto, T Nagasaka, A Nawa, S Nomura, F Kikkawa

**Affiliations:** 1 Department of Obstetrics and Gynecology, Nagoya University Graduate School of Medicine, 65 Tsurumai-cho, Showa-ku, Nagoya 466-8550, Japan; 2 Division of Pathology/Clinical Laboratory, Nagoya University Graduate School of Medicine, Nagoya, Japan

**Keywords:** angiotensin II, AT_1_ receptor, angiogenesis, ovarian cancer, VEGF, prognosis

## Abstract

Angiotensin II, a main effector peptide in the renin–angiotensin system, acts as a growth-promoting and angiogenic factor via type 1 angiotensin II receptors (AT_1_R). We have recently demonstrated that angiotensin II enhanced tumour cell invasion and vascular endothelial growth factor (VEGF) secretion via AT_1_R in ovarian cancer cell lines *in vitro*. The aim of the present study was to determine whether AT_1_R expression in ovarian cancer is correlated with clinicopathological parameters, angiogenic factors and patient survival. Immunohistochemical staining for AT_1_R, VEGF, CD34 and proliferating cell nuclear antigen (PCNA) were analysed in ovarian cancer tissues (*n*=67). Intratumour microvessel density (MVD) was analysed by counting the CD34-positive endothelial cells. Type 1 angiotensin II receptors were expressed in 85% of the cases examined, of which 55% were strongly positive. Type 1 angiotensin II receptors expression was positively correlated with VEGF expression intensity and MVD, but not with histological subtype, grade, FIGO stage or PCNA labelling index. In patients who had positive staining for AT_1_R, the overall survival and progression-free survival were significantly poor (*P*=0.041 and 0.017, respectively) as compared to those in patients who had negative staining for AT_1_R, although VEGF, but not AT_1_R, was an independent prognostic factor on multivariate analysis. These results demonstrated that AT_1_R correlated with tumour angiogenesis and poor patient outcome in ovarian cancer, suggesting its clinical potential for a novel molecular target in strategies for ovarian cancer treatment.

Ovarian cancer is the leading cause of death from female genital malignancies despite significant advances in diagnosis and treatment (McGuire and Markman, 2003). Several clinicopathological parameters have been reported to be of prognostic significance in ovarian cancer, including disease stage, histological grade, histological subtype, residual tumour volume, presence of cytologically malignant ascites and response to chemotherapy (Omura *et al*, 1991; Chi *et al*, 2001). In addition to these established clinical parameters, the identification of biochemical or molecular markers more strictly related to intrinsic tumour cell behaviour in ovarian cancer and its characteristic progression pathway may be helpful in improving the survival of patients with this disease.

Angiotensin II, a multifunctional bioactive peptide in the renin–angiotensin system (RAS), plays a fundamental role in controlling cardiovascular and renal homeostasis. Angiotensin II also acts as a potent growth factor not only for vascular smooth muscle cells, but also for certain cancer cell lines (Fujimoto *et al*, 2001; Muscella *et al*, 2002). In addition, angiotensin II stimulates angiogenesis via the upregulation of vascular endothelial growth factor (VEGF) (Le Noble *et al*, 1993; Chua *et al*, 1998; Pupilli *et al*, 1999; Tamarat *et al*, 2002). These cellular effects of angiotensin II are mostly mediated through specific G-protein-coupled AT_1_R.

Recent studies have proposed the concept of a localized tissue RAS in various organs (Nielsen *et al*, 1995), and activation of the RAS has been demonstrated under neoplastic conditions (Inwang *et al*, 1997; Takeda and Kondo, 2001; Juillerat-Jeanneret *et al*, 2004). In the tumour-related RAS, angiotensin II is abundantly generated from angiotensin I by angiotensin-converting enzyme (ACE), and AT_1_R expression is generally upregulated. Previous studies showed that the angiotensin II–AT_1_R system is deeply involved in tumour growth, metastasis and angiogenesis in experimental animal models, suggesting a therapeutic potential of RAS blockade using an ACE inhibitor or AT_1_R blocker (Rivera *et al*, 2001; Yoshiji *et al*, 2001; Fujita *et al*, 2002; Miyajima *et al*, 2002; Egami *et al*, 2003; Uemura *et al*, 2003; Arrieta *et al*, 2005).

In gynaecological malignancies, prior studies at our laboratory demonstrated that angiotensin II stimulated *in vitro* cell proliferation, invasion, or VEGF secretion via AT_1_R in cervical cancer (Kikkawa *et al*, 2004; Suganuma *et al*, 2004), endometrial cancer (Watanabe *et al*, 2003) and choriocarcinoma (Ino *et al*, 2003). Recently, we were the first to show that AT_1_R is expressed in human ovarian cancer cells and angiotensin II enhanced tumour cell invasion and VEGF expression/secretion via AT_1_R (Suganuma *et al*, 2005). Furthermore, we demonstrated that AT_1_R blocker suppresses angiogenesis and the peritoneal dissemination of ovarian cancer in a mouse model (Suganuma *et al*, 2005). These results prompted us to hypothesize that angiotensin II acts as an angiogenic and tumour-progressive factor for ovarian cancer, and that AT_1_R may have clinical potential as a novel molecular target or as a prognostic indicator in the treatment of ovarian cancer, as well as in other gynaecological malignancies.

Based on these findings, the present study examined the immunohistochemical expression of AT_1_R in ovarian cancer tissues to determine whether AT_1_R expression is correlated with clinicopathological factors or angiogenic parameters, including VEGF expression and intratumour microvessel density (MVD). Furthermore, we assessed whether AT_1_R correlates with the prognosis of ovarian cancer patients.

## MATERIALS AND METHODS

### Reagents and antibodies

Rabbit polyclonal antibody against AT_1_R (306) was purchased from Santa Cruz Biotechnology (Santa Cruz, CA, USA). Rabbit anti-human VEGF polyclonal antibody (A-20) was also purchased from Santa Cruz. Mouse monoclonal antibody against human CD34, a marker of endothelial cells, was obtained from Immunotech (Marseille, France). Antiproliferating cell nuclear antigen (PCNA) monoclonal antibody PC10 was purchased from Dako (Glostrup, Denmark).

### Patients and tissue samples

Human epithelial ovarian cancer tissues (*n*=67) were obtained from patients who underwent surgical treatment at Nagoya University Hospital between 1993 and 2002. Surgical treatment consisted of total abdominal hysterectomy and bilateral salpingo-oophorectomy, followed by surgical staging and/or debulking surgery (if necessary). All tissue samples were fixed in 10% formalin, embedded in paraffin, and routinely stained with haematoxylin and eosin for histological examination. The histological cell types and histological grade (tumour differentiation) were assigned according to the criteria of the World Health Organization (WHO) classification. Clinical staging was reviewed based on the International Federation of Gynecology and Obstetrics (FIGO) staging system. The patients received postoperative chemotherapy with platinum plus cyclophosphamide and doxorubicin (before 1997), or platinum plus paclitaxel (after 1997) for high-risk early stage (stage I with grade 3; stage IC; any stage II) or advanced diseases (stages III and IV). Tumour recurrence/progression was defined based on clinical, radiological or histological diagnosis. The use of tissue samples was approved by the Institutional Review Board (IRB) of Nagoya University Graduate School of Medicine and by individual patients, respectively.

### Immunohistochemistry

Formalin-fixed, paraffin-embedded tissue sections were cut at a thickness of 4 *μ*m. For heat-induced epitope retrieval, deparaffinized sections in 0.01 M citrate buffer (Target Retrieval Solution pH 6.1, Dako) were treated three times at 90°C for 5 min using a microwave oven. Immunohistochemical staining was performed using the avidin–biotin immunoperoxidase technique (Histofine SAB-PO kit, Nichirei, Tokyo, Japan). Endogenous peroxidase activity was blocked by incubation with 0.3% H_2_O_2_ in methanol for 15 min, and nonspecific immunoglobulin binding was blocked by incubation with 10% normal goat serum for 10 min. Sections were incubated at room temperature for 2 h with primary antibody (anti-AT_1_R at 1 : 100 dilution, anti-VEGF at 1 : 200, anti-CD34 at 1 : 40, anti-PCNA at 1 : 40). The sections were rinsed and incubated for 30 min with biotinylated second antibody. After washing, the sections were incubated for 30 min with horseradish peroxidase-conjugated streptavidin, and finally treated with 3-amino-9-ethylcarbazole (AEC) in 0.01% H_2_O_2_ for 10 min. The slides were counterstained with Meyer's haematoxylin. As a negative control, the primary antibody was replaced with normal rabbit IgG or mouse IgG at an appropriate dilution. As positive immunohistochemical controls, marked immunoreactivity of AT_1_R and VEGF in placental trophoblastic tissues was confirmed as reported previously (Shiraishi *et al*, 1996; Ino *et al*, 2003). The immunostaining intensity for AT_1_R and VEGF was scored semiquantitatively based on the percent positivity of stained cells on a three-tiered scale as follows: −, negative (no positive cells); +, focally or weakly positive (<50% positive cells); ++, diffusely or strongly positive (>50% positive cells). In each case, at least three different areas were evaluated and the mean of the results was considered to be the expression intensity score. The staining procedure for each antibody was repeated twice, and it was confirmed that there was no difference in the staining intensity between the two experiments. The scoring procedure was carried out twice by two independent observers (each blinded to the other's score) without any knowledge of the clinical parameters or other prognostic factors. The concordance rate was over 95% between the observers. In the case of disagreement, the slides were reviewed simultaneously by these two observers, with another, different observer, who were seated together at a multiheaded microscope in order to resolve the difference of opinion.

### Evaluation of tumour angiogenesis and proliferation

Tumour angiogenesis was assessed by counting the CD34-positive capillaries and small venules, according to the method of Weidner *et al* (1992). First, after scanning the immunostained section under a light microscope at low magnification (× 40 and × 100), the area within the tumour having the highest number of distinct CD34-staining microvessels (‘hot spots’) was selected. For the determination of intratumour MVD, all microvessels were counted within the neovascular hot spot under a light microscope at × 200 magnification (× 20 objective lens and × 10 ocular lens). Two investigators independently evaluated tumour vascularity without any information about the clinicopathological features or expression scores for AT_1_R and VEGF. The average number of microvessels of the three × 200 fields that were strictly confined to the hot spot area was recorded as the MVD, as described previously (Ueda *et al*, 1999).

To assess proliferation, sections were immunostained with the cellular proliferation marker PCNA as described previously (Thomas *et al*, 1995; Fujimura *et al*, 2000). The PCNA labelling index (PCNA LI) was defined as the number of tumour cells with nuclear PCNA immunostaining divided by the total number of tumour cells, and expressed as a percentage. A total of 1000 nuclei in the selected area were counted under a light microscope at high magnification (× 400 fields) and the mean percentages were recorded as the PCNA LI.

### Statistical analysis

Spearman's rank correlation test was performed to analyse the correlation between AT_1_R expression scores and various parameters. Fisher's exact test or chi-square (*χ*^2^) test were also used to analyse the distribution of AT_1_R-strongly positive cases, according to clinicopathological, angiogenic and proliferative features.

Overall survival (OS) was calculated from the date of diagnosis to the date of death, and progression-free survival (PFS) was calculated from the date of diagnosis to the date of progression/recurrence or date of last follow-up. Survival analyses were performed according to the life tables method and according to the Kaplan–Meier method. Comparison of the survival between groups was performed with the log-rank test. Cox proportional-hazard analysis was used for univariate and multivariate analysis to explore the effect of variables on survival. The SAS software (SAS Institute Inc., Cary, NC, USA) was used for all statistical analyses and a *P*-value of <0.05 was considered significant.

## RESULTS

### Immunohistochemical expression of AT_1_R in ovarian cancer tissues

**Figure 1:**
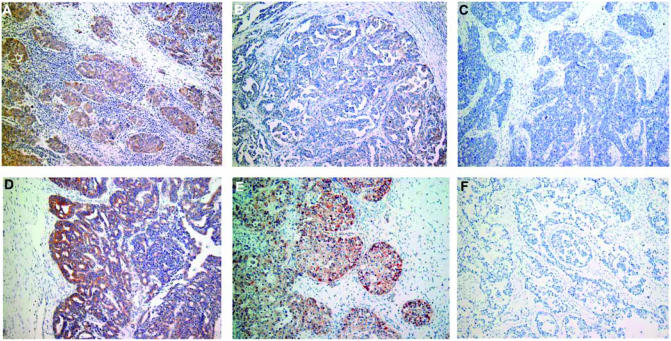
Representative immunohistochemical staining for AT_1_R in human ovarian cancer tissues. (**A**) Strongly positive; (**B**) weakly positive; (**C**) negative in serous adenocarcinoma; (**D**) strongly positive in endometrioid adenocarcinoma; (**E**) strongly positive in clear cell adenocarcinoma; (**F**) negative control. Original magnification, × 100 in (**A**–**F**).

As shown in Figure 1, the immunoreactivity of AT_1_R was detected at variable levels, and was localized both on the membrane and in the cytoplasm of tumour cells. There was no immunoreactivity of AT_1_R in the tumour stroma. Of the 67 ovarian cancer specimens examined in this study, AT_1_R was detected in 57 (85%) cases, of which 37 (55%) were strongly positive. AT_1_R-negative tumours were found in only 10 cases (15%).

### Correlation of AT_1_R expression with clinicopathological, angiogenic and proliferative parameters

**Figure 2:**
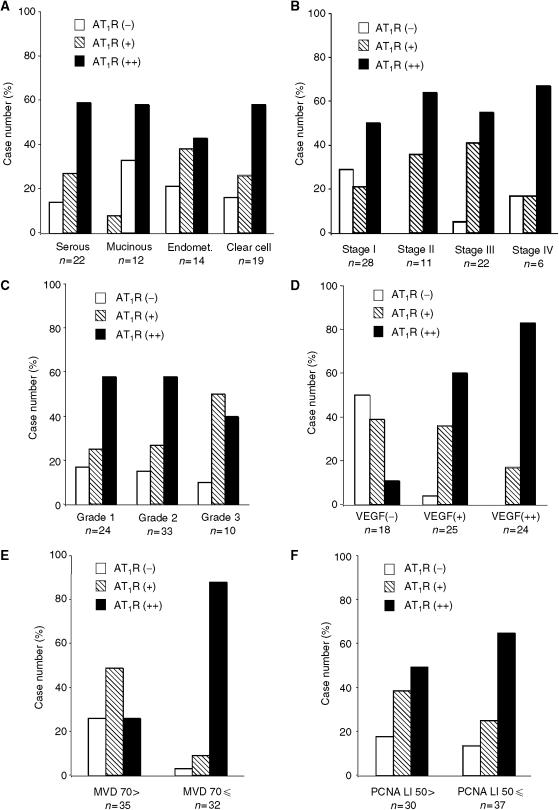
Correlation of AT_1_R expression intensity with histological subtype (**A**), FIGO stage (**B**), histological grade (**C**), VEGF expression intensity (**D**), MVD number (**E**), and PCNA LI (**F**) in ovarian cancer tissues. Significant correlation (*P*<0.05) was observed between AT_1_R expression and VEGF expression (**D**), or between AT_1_R expression and MVD number (**E**), while there was no significant correlation between AT_1_R expression and other clinicopathological factors or PCNA LI.

The correlation of AT_1_R expression with clinicopathological, angiogenic and proliferative parameters was analysed in 67 ovarian cancer tissues. Type 1 angiotensin II receptors expression did not significantly correlate with the histological subtype, FIGO stage or histological grade (tumour differentiation) (Figure 2A–C). In contrast, the AT_1_R expression positively correlated with the VEGF staining scores (Figure 2D). Of the VEGF-strongly positive cases (*n*=24), 20 (83%) were AT_1_R strongly positive, while half (*n*=9) of the VEGF-negative cases (*n*=18) were AT_1_R negative (Figure 2D).

To determine the correlation between AT_1_R expression and tumour angiogenesis, we assessed the intratumour MVD by counting CD34-positive microvessels in the same series of ovarian cancer tissues (*n*=67). Microvessel density ranged broadly from 20 to 150 (mean=75.2, median=68). Thus, we defined cases with MVD number of more than 70 as high MVD (*n*=32), while cases with MVD number of <70 were defined as low MVD (*n*=35). Interestingly, the AT_1_R expression score positively correlated with MVD, and 28 (87.5%) of 32 high MVD cases were AT_1_R strongly positive (Figure 2E). In this study, we confirmed that the MVD number did not correlate with the histological subtype, FIGO stage or histological grade, while VEGF expression scores positively correlated with the MVD number (data not shown). Taken together, our findings indicate that AT_1_R, as well as VEGF, are associated with tumour angiogenesis of ovarian carcinoma.

Next, we assessed the correlation between AT_1_R expression and tumour proliferation using immunohistochemical staining with PCNA in the same series of ovarian cancer tissues. The PCNA LI ranged from 16 to 80%, with a mean value of 50.16%, which is well consistent with the results from a previous report (Thomas *et al*, 1995). We defined cases with a PCNA LI of more than 50 as high PCNA LI (*n*=37), while cases with a PCNA LI of <50 were defined as low PCNA LI (*n*=30). As shown in Figure 2F, the AT_1_R expression score did not significantly correlate with PCNA LI.

The results from correlation analyses of AT_1_R overexpression (strongly positive cases) with clinicopathological parameters, VEGF expression, MVD and PCNA are summarized in Table 1. These analyses demonstrated that AT_1_R overexpression was significantly correlated with VEGF overexpression and high MVD number, but not with the proliferation marker PCNA, nor any of the clinicopathological factors examined in this study.

### Correlation of AT_1_R expression with survival of ovarian cancer patients

Follow-up data were available for 58 patients (nine patients were lost to follow-up). The median follow-up was 60 months (range 1–121 months). During the follow-up period, the total number of cases in which death and progression/recurrence were observed was 19 (32.8%) and 25 (43.1%), respectively.

**Figure 3:**
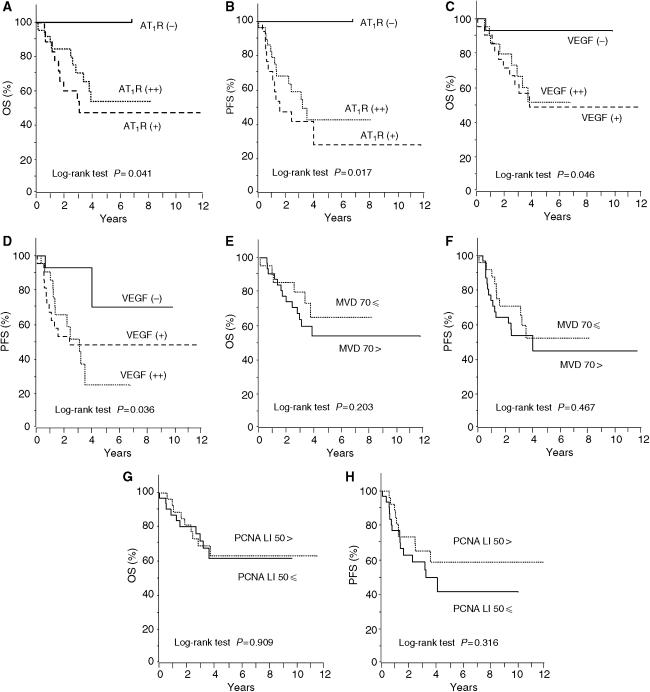
Overall survival (OS) and progression-free survival (PFS) curves drawn using the Kaplan–Meier method according to the AT_1_R expression (**A** and **B**), VEGF expression (**C** and **D**), MVD number (**E** and **F**), and PCNA LI (**G** and **H**). Significant differences in the OS and PFS according to the AT_1_R expression (*P*=0.041 and 0.017, respectively), and according to the VEGF expression (*P*=0.046 and 0.036, respectively). No significant difference in the OS and PFS according to the MVD (*P*=0.203 and 0.467, respectively) and according to the PCNA LI (*P*=0.909 and 0.316, respectively).

To evaluate the impact of AT_1_R expression on patient prognosis, overall survival (OS) and PFS curves were constructed using the Kaplan–Meier method. The 5-year OS rates of patients who had negative (*n*=10), weakly positive (*n*=18), and strongly positive (*n*=30) expression for AT_1_R were 100, 45.8 and 55.7%, respectively (Figure 3A). The 5-year PFS rates of patients who had negative, weakly positive, and strongly positive expression for AT_1_R were 100, 27.5 and 41.9%, respectively (Figure 3B). Both OS and PFS in patients with positive (weak and strong) expression for AT_1_R were significantly lower than those in patients with negative AT_1_R expression (*P*=0.041 and 0.017, respectively, by log-rank test). However, there was no significant difference in the distributions of OS and PFS between the AT_1_R-weakly positive and AT_1_R-strongly positive groups.

The correlation of VEGF expression, intratumour MVD, and PCNA LI with prognosis was also analysed. The 5-year OS rates of patients who had negative (*n*=15), weakly positive (*n*=22) and strongly positive (*n*=21) expression for VEGF were 92.9, 48.4 and 51.3%, respectively (Figure 3C). The 5-year PFS rates of patients who had negative, weakly positive and strongly positive expression for VEGF were 69.6, 47.6 and 24.2%, respectively (Figure 3D). Both OS and PFS in patients with positive expression for VEGF were significantly lower than those in patients with negative VEGF expression (*P*=0.046 and 0.036, respectively, by log-rank test), although there was no significant difference in the distributions of OS and PFS between the VEGF-weakly positive and strongly positive groups. In contrast to AT_1_R and VEGF, there was no significant difference in the distributions of OS and PFS according to the MVD number (*P*=0.203 and 0.467, respectively), or according to the PCNA LI (*P*=0.909 and 0.316, respectively), as shown in Figure 3E–H. These results indicate that AT_1_R and VEGF, but not the MVD number and PCNA LI, significantly correlated with the impaired survival of ovarian cancer patients.

### Multivariate analysis of prognostic variables in ovarian cancer patients

Cox proportional-hazard analysis was performed to determine the impact of various factors on survival. The results of univariate/multivariate analyses of the variables, including AT_1_R, VEGF, MVD and PCNA LI, with respect to OS and PFS are shown in Tables 2 and 3, respectively. Because none of the patients with negative AT_1_R expression (*n*=10) died or recurred (no risk for OS and PFS), the univariate analysis for AT_1_R expression (negative or weakly/strongly positive) could not be performed, and it was not entered into the multivariate analysis model. Among the other six variables, the FIGO stage (*P*=0.025) and VEGF expression (*P*=0.018) were statistically significant prognostic factors with respect to OS on multivariate analysis (Table 2). Similarly, the FIGO stage (*P*=0.015) and VEGF expression (*P*=0.008) were found to be independent prognostic factors with respect to PFS on multivariate analysis (Table 3).

## DISCUSSION

In the present study, we demonstrated the expression of the type 1 angiotensin II receptor, AT_1_R, in human ovarian carcinoma tissues, and its significant correlation with tumour angiogenesis and patient survival. It has been reported that AT_1_R is expressed in various human malignant tumour tissues, including breast cancer (Inwang *et al*, 1997), skin cancer (Takeda and Kondo, 2001), pancreatic cancer (Fujimoto *et al*, 2001), laryngeal carcinoma (Marsigliante *et al*, 1996) and prostate cancer (Uemura *et al*, 2003). Furthermore, we recently demonstrated that AT_1_R was expressed in gynaecological malignancies, including cervical cancer (Kikkawa *et al*, 2004), endometrial cancer (Watanabe *et al*, 2003), choriocarcinoma (Ino *et al*, 2003), and ovarian cancer (Suganuma *et al*, 2005). These findings suggest that AT_1_R exists in a wide spectrum of human cancers, especially in gynaecological malignancies, and that the angiotensin II-AT_1_R system may play significant roles in the localized RAS within these tumour tissues. Evidence for the involvement of AT_1_R in tumour progression, such as growth, metastasis and angiogenesis, has accumulated in various animal models (Rivera *et al*, 2001; Fujita *et al*, 2002; Miyajima *et al*, 2002; Egami *et al*, 2003; Uemura *et al*, 2003; Arrieta *et al*, 2005). Until now, however, there have been no reports analysing the correlation of AT_1_R expression with clinical parameters, especially with patient prognosis, using a large scale of clinical samples of human cancers. Thus, the present study is the first to investigate the correlation between AT_1_R and clinical outcome in ovarian cancer patients.

Our immunohistochemical analysis showed that AT_1_R was present in 85% of ovarian carcinomas examined, and it was overexpressed in more than half (55%) of the cases. Furthermore, AT_1_R expression was not dependent on the histological subtype, grade or FIGO stage, although the rate of AT_1_R-negative cases was relatively high in FIGO stage I (eight of 28 cases: 29%) as compared to those in FIGO II–IV. Our prior studies showed that AT_1_R expression was almost absent in benign ovarian cystadenoma, but was dramatically upregulated with progression from borderline malignancy to invasive ovarian carcinoma (Suganuma *et al*, 2005). These results suggest the possible involvement of AT_1_R in the specific cell behaviours that were common to the malignant phenotypes, such as tumour cell invasion or aggressive neovascularization, rather than in the degree of tumour differentiation or histopathological subtypes.

It is of particular interest that AT_1_R expression was positively correlated with VEGF expression intensity in the ovarian carcinomas examined in this study. Vascular endothelial growth factor is known to be the main angiogenic factor in ovarian cancer (Yamamoto *et al*, 1997; Fujimoto *et al*, 1998; Bamberger and Perrett, 2002). It has been shown that angiotensin II upregulated VEGF expression via AT_1_R (Chua *et al*, 1998; Pupilli *et al*, 1999; Tamarat *et al*, 2002), and this angiotensin II-induced VEGF upregulation was mediated by hypoxia-inducible factor-1, even in non-hypoxic conditions (Richard *et al*, 2001). We have recently reported that angiotensin II stimulated VEGF expression and secretion in AT_1_R-positive ovarian cancer cells *in vitro* (Suganuma *et al*, 2005). Furthermore, we found that ACE, an enzyme producing angiotensin II in the RAS, was expressed in the tumour stroma of ovarian cancer (data not shown). Thus, the positive correlation between AT_1_R and VEGF observed in the present study not only reflects the results from the prior *in vitro* studies, but also suggests the existence of the angiotensin II-AT_1_R-VEGF system controlling angiogenic signals in ovarian cancer.

In addition to VEGF, the intratumour MVD number also positively correlated with AT_1_R expression intensity. Indeed, AT_1_R was overexpressed in 87.5% of the cases with high MVD numbers (more than 70). It is well known that MVD is a most reliable tool for reflecting tumour angiogenesis (Weidner *et al*, 1992) and it has been reported that VEGF expression directly correlated with increased MVD in a variety of tumours (Toi *et al*, 1996; Maeda *et al*, 1996). In the present study, we found the positive correlation of MVD number not only with VEGF, but also with AT_1_R. These findings support our hypothesis that AT_1_R is a key molecule in tumour angiogenesis in ovarian cancer.

It has been reported that AT_1_R is involved in the proliferation of various cancers *in vitro* and *in vivo* (Fujimoto *et al*, 2001; Rivera *et al*, 2001; Muscella *et al*, 2002; Ino *et al*, 2003; Uemura *et al*, 2003). However, our immunohistochemical study showed that the AT_1_R expression score did not significantly correlate with the positivity of the cellular proliferation marker PCNA in ovarian cancer tissues. Consistently, we previously reported that angiotensin II enhanced the invasive activity and VEGF secretion, but not cell proliferation, in AT_1_R-positive ovarian cancer cell lines *in vitro* (Suganuma *et al*, 2005). Taken together, it appears that AT_1_R is not directly involved in tumour cell proliferation in ovarian cancer, and that the angiotensin II-AT_1_R system plays differential roles in cancer progression, which may be dependent upon tumour type.

Our results from survival analyses demonstrated that both OS and PFS were significantly poorer in AT_1_R-positive cases than those in AT_1_R-negative cases. This provides the first evidence for the positive correlation of AT_1_R expression with poor clinical outcome in human malignancies. Surprisingly, we observed 100% of the 5-year OS and PFS in AT_1_R-negative cases, although the case number of these AT_1_R-non-expressing ovarian carcinomas was small (*n*=10) in this study. In addition, we showed that there was no significant difference in the survival between AT_1_R strongly positive and weakly positive within the AT_1_R-expressing cases. These results might be unexpected because the immunohistochemical analysis showed a clear correlation between overexpression (strongly positive) of AT_1_R with high VEGF expression and high MVD numbers. One could speculate that the existence (either high or low expression) of AT_1_R plays a crucial role in the initiation of angiogenic signals in the primary lesions of ovarian cancer, while other various molecules are complexly involved in subsequent tumour progression, such as peritoneal dissemination or distal metastasis. Similar to AT_1_R, both the OS and PFS in VEGF-positive cases were significantly poorer than those in VEGF-negative cases, which is consistent with the observation in previous studies (Yamamoto *et al*, 1997; Shen *et al*, 2000). These findings suggest that AT_1_R, as well as VEGF, may become a prognostic marker for ovarian cancer patients; however, our multivariate analyses showed that FIGO stage and VEGF, but not AT_1_R, were independent prognostic factors for both OS and PFS. These results may be due, at least in part, to the small number of patient samples, because the AT_1_R-negative group consisted of only 10 patients, and there was no death or recurrence in this group. Further studies, including multivariate analyses using a larger sample size, are required to clarify whether AT_1_R can be an independent prognostic factor or not.

In contrast to AT_1_R and VEGF, our study demonstrated that there was no significant difference in the survival according to the MVD number or PCNA LI. Previous studies showed that MVD was not a useful prognostic factor in ovarian cancer (Obermair *et al*, 1999; Shen *et al*, 2000; Bamberger and Perrett, 2002). On the other hand, Hollingsworth *et al* (1995) reported that MVD was significantly correlated with poor prognosis of advanced stage ovarian cancer, while conversely it was associated with better prognosis (Ogawa *et al*, 2002). Correlation of the cellular proliferation marker index, such as PCNA and Ki-67, with patient survival is also controversial in ovarian cancer (Nakopoulou *et al*, 1993; Thomas *et al*, 1995; Itamochi *et al*, 2002). The reason for these controversial findings remains unclear, but may be due at least in part to the complicated mechanisms for tumour angiogenesis and growth of ovarian cancer that are differentially regulated among the histological subtypes (Bamberger and Perrett, 2002).

In summary, we demonstrated that AT_1_R expression was associated with tumour angiogenesis of ovarian cancer and also correlated with poor patient outcome. There has been accumulated evidence that AT_1_R blockade therapy suppresses tumour growth, metastasis and angiogenesis in experimental animal models (Rivera *et al*, 2001; Fujita *et al*, 2002; Miyajima *et al*, 2002; Egami *et al*, 2003; Uemura *et al*, 2003; Arrieta *et al*, 2005), and we also demonstrated that an AT_1_R blocker suppressed the peritoneal dissemination of ovarian cancer in a mouse model (Suganuma *et al*, 2005). Combined with these findings, the present data suggest that AT_1_R has clinical potential not only as a prognostic indicator, but also as a novel molecular target in strategies for ovarian cancer treatment.

## Figures and Tables

**Table 1:** Correlation of AT_1_R overexpression with clinicopathologic factors, and angiogenesis/proliferation markers in ovarian cancer

	**Patients**		**AT_1_R overexpression**		
	**No.**	**%**	**No.**	**%**	***P*-value**
All cases	67	100.0	37	55.2	
					
*Histological type*					
Serous	22	32.8	13	59.1	0.777^a^
Mucinous	12	17.9	7	58.3	
Endometrioid	14	20.9	6	42.9	
Clear cell	19	28.4	11	57.9	
					
*Histological grade*					
G1	24	35.8	14	58.3	0.576^a^
G2	33	49.3	19	57.6	
G3	10	14.9	4	40.0	
					
FIGO stage					
I	28	41.8	14	50.0	0.814^a^
II	11	16.4	7	63.6	
III	22	32.8	12	54.5	
IV	6	9.0	4	66.7	
					
*VEGF expression*					
(−)	18	26.9	2	11.1	0.001^b^
(+)	49	73.1	35	71.4	
					
*Microvessel density*					
70>	35	52.2	9	25.7	0.001^b^
70⩽	32	47.8	28	87.5	
					
*PCNA LI*					
50>	30	44.8	14	46.7	0.205^b^
50⩽	37	55.2	23	62.2	

a *χ*^2^ test.

b Fisher's exact test.

**Table 2:** Univariate and multivariate analyses of overall survival (OS) in ovarian cancer patients

		**Univariate analysis**			**Multivariate analysis**		
**Variables**	**Categories**	**Hazard ratio**	**95% CI**	***P*-value**	**Hazard ratio**	**95% CI**	***P*-value**
FIGO stage	I/II	2.60	1.00–6.72	0.049	2.96	1.15–4.61	0.025
	III/IV						
							
Histological grade	1 or 2	0.19	0.02–1.56	0.122	0.22	0.03–1.71	0.147
	3						
							
AT1R expression	(−)	ND^a^	ND^a^	ND^a^	—	—	—
	(+)/(++)						
							
AT1R overexpression	(−)/(+)	0.86	0.25–2.98	0.815	—	—	—
	(++)						
							
VEGF expression	(−)	5.75	0.71–46.52	0.101	12.18	1.54–96.37	0.018
	(+)/(++)						
							
MVD	<70	0.27	0.09–0.77	0.145	0.30	0.11–0.84	0.122
	⩾70						
							
PCNA LI	<50	1.13	0.44–2.88	0.805	—	—	—
	⩾50						

CI=confidence interval.

a ND=not determined because no patients with negative AT1R expression died or recurred.

**Table 3:** Univariate and multivariate analyses of progression-free survival (PFS) in ovarian cancer patients

		**Univariate analysis**			**Multivariate analysis**		
**Variables**	**Categories**	**Hazard ratio**	**95% CI**	***P*-value**	**Hazard ratio**	**95% CI**	***P*-value**
FIGO stage	I/II	2.29	1.02–5.14	0.044	2.70	1.21–6.04	0.015
	III/IV						
							
Histological grade	1 or 2	0.58	0.16–2.08	0.400	—	—	—
	3						
							
AT1R expression	(−)	ND^a^	ND^a^	ND^a^	—	—	—
	(+)/(++)						
							
AT1R overexpression	(−)/(+)	0.85	0.26–2.75	0.789	—	—	—
	(++)						
							
VEGF expression	(−)	4.10	0.88–19.02	0.072	7.49	1.68–33.32	0.008
	(+)/(++)						
							
MVD	<70	0.36	0.15–0.88	0.125	0.46	0.20–1.06	0.096
	⩾70						
							
PCNA LI	<50	1.49	0.65–3.42	0.343	—	—	—
	⩾50						

CI=confidence interval.

a ND=not determined because no patients with negative AT1R expression died or recurred.
